# Analysis of health claims regarding creatine monohydrate present in commercial communications for a sample of European sports foods supplements

**DOI:** 10.1017/S1368980020005121

**Published:** 2021-01-20

**Authors:** Lucía Molina Juan, Isabel Sospedra, Alejandro Perales, Cristina González-Díaz, Angel Gil-Izquierdo, José Miguel Martínez-Sanz

**Affiliations:** 1 Faculty of Health Sciences, University of Alicante, Alicante, Spain; 2 Nursing Department, Faculty of Health Sciences, University of Alicante, Campus de Sant Vicent Del Raspeig, Ap.99, E-03080 Alacant, Spain; 3 Communication Sciences and Sociology, Faculty of Communication Sciences, Rey Juan Carlos University, Madrid, Spain; 4 Psychology and Social Communication Department, Faculty of Economics and Business, University of Alicante, Alacant, Spain; 5 Quality, Safety, and Bioactivity of Plant Foods Group, Department of Food Science and Technology, CEBAS-CSIC, University of Murcia, Murcia, Spain

**Keywords:** Nutrition, Sports performance, Creatine, Health claims, Nutritional labelling

## Abstract

**Objective:**

To analyse the information on health claims present in the labelling of creatine monohydrate (CM) products.

**Design:**

A descriptive study of a selection of products marketed as CM, with health claims, and that met the inclusion/exclusion criteria, was conducted using the Amazon and Google Shopping websites. The adequacy and compliance of the health claims were evaluated with the European legislative requirements (European Food Safety Authority and European Commission). The results were discussed with scientific evidence criteria from the Academy of Nutrition and Dietetics, International Olympic Committee, and International Society of Sports Nutrition, as well as the systematic review carried out by Mielgo-Ayuso in 2019.

**Setting:**

Health claims in the commercial communications of a sample of CM supplements, in relation to current legislation and scientific knowledge.

**Participant:**

A total of 554 CM products were obtained.

**Results:**

Of the total sample, only 167 met the inclusion/exclusion criteria. Of these, 30·5 % recommended a CM dose of 5·0–5·9 g/d, while 29·9 % recommended 3·0 to 3·9 g/d. As for the health claims, ‘Enhances physical performance’ appeared in 73·1 % of the supplements, in most cases referring to a dosage of 3·0 to 3·9 g/d for these products. The rest of the declarations were not adequate or needed to be modified.

**Conclusion:**

Only 25 % of the health claims complied with the criteria established by the scientific reference documents. Most of the declarations must be modified or eliminated, as they could be considered fraudulent and/or misleading for the consumer.

## Sports foods supplements

Ergogenic aids refer to the application of any method or manoeuvre to improve the ability to carry out a certain physical work or sports performance^(1)^. Among the ergogenic aids, different types can be differentiated: mechanical, physiological, pharmacological, psychological and nutritional^(2)^. Ergonomic aids or sports foods supplements are, according to the International Olympic Committee (IOC), ‘a food, component, nutrient or non-food component that is purposely ingested within the normal diet with the aim of obtaining a determined effect on health or performance’^(3)^. Currently, they are used by a large portion of athletes, both professional and amateur, to improve their performance^(4)^. These substances are consumed due to the existence of competitions in which athletes aim to achieve certain objectives related to sports performance^(3)^.

## Sports food supplements: scientific evidence and legislation

Although a multitude of sports foods supplements are currently marketed, studies on their effects highlight that scientific evidence showing that they promote health benefits or improve the sports performance of the athlete is only available for a few of them^(5)^. Creatine, caffeine, bicarbonate, β-alanine, carbohydrates and proteins are the sports food supplements that stand out due to the high number of studies documenting their effects, both through laboratory research and the positive response of athletes^(4,6)^. Scientific organisations and public institutions such as the European Food Safety Authority (EFSA) have previously studied the characteristics of the different substances added to or isolated from supplements, as well as the safety of their consumption; among these substances, creatine monohydrate (CM) stands out^(7)^.

In addition to the importance of scientific evidence for the approval of the consumption of a certain sports supplement, the presence of legislation that regulates the marketing and advertising of products aimed at the consumer must also be highlighted. At the European level, sports food supplements, such as CM, are covered by Regulation (EC) No. 1924/2006 regarding nutritional and health claims for food; Regulation (EU) nº 1169/2011 on food information provided to the consumer; Regulation (EC) 258/97 on novel foods and novel food ingredients and Directive 2002/46 on the approximation of the laws of the Member States regarding food supplements^(9)^.

## Creatine monohydrate

Creatine is a nitrogenous compound that is available from various foods in a normal diet, and it serves as an energy substrate for skeletal muscle contraction. The intention of supplementation with this substance is to increase the levels of phosphocreatine at rest, as well as those of free creatine, with the aim of reducing muscle fatigue, which is important in the search for better results in sporting events^(10)^. Muscle creatine concentrations are increased by 20 % with CM supplementation^(11)^. These supplements increase lean body mass, as well as strength, power and effectiveness in short-duration, high-intensity exercises^(12)^. The increase in body mass may be a result of the increase in intracellular water related to the osmotic properties of creatine^(13)^. Studies on CM supplementation have shown short-duration improvements in sports performance and strength: specifically, in maximum-intensity exercises, muscle power, number of repetitions, muscle endurance, speed and total strength^(10)^.

The use of CM can yield increases in power during short sprints of maximum intensity, which can be even more evident when repeated sprints are accompanied by short recovery periods. Furthermore, with CM supplementation, effects are also observed in muscle glycogen stores^(13)^. This is important because the availability of muscle glycogen is the main determinant of sports performance in resistance exercises, and its depletion can lead to muscle fatigue^(4)^. In addition, CM is one of the few sports foods supplements or ergonomic aids with health claims authorised by the EFSA and the European Commission (EC), due to its evident effects on the health and sports performance of athletes^(14)^. The approved health claims are ‘Creatine increases physical performance in repeated bursts of high-intensity exercise in the short term’ and ‘Daily consumption of Creatine may improve the effect of resistance training on muscle strength in adults over 55 years of age’. These health claims refer to the 3-g dose of CM^(15)^. There are also other institutions that describe the use of sports foods supplements, such as the Academy of Nutrition and Dietetics (AND)^(16)^, based on the evidence studied by Tarnopolsky^(17)^ and the IOC^(3)^.

These health claims occupy a pivotal position in the marketing of the product, both in its labelling and in the advertising of the product intended for athletes^(18)^. There have been cases in which the health claims did not fully match the accurate descriptions of the effects that sports foods supplements produce on the health of users who consume them^(19)^. Therefore, the aims of this study are to describe the health claims for CM present in commercial messages for a sample of sports foods supplements and to verify the adequacy of these health claims according to current European regulations.

## Methods

To achieve the proposed aims, we carried out a descriptive study to analyse the contents of the different health claims included in the commercial communications of a sample of sports foods supplements, by analysing these claims in light of current legislation and scientific evidence in this area.

First, we have performed a review of the current legislation and scientific evidence in this area. Second, we analysed the contents of the different health claims included in the commercial communications of a sample of CM supplements. Lastly, we observed if the health claims were in compliance with current European legislation and the review of research studies.

### Population selection strategy

The search for the sample products was carried out in April 2019, through the Amazon and Google Shopping web portals. The term ‘Creatine Monohydrate’ was introduced and filtered by country or European region to carry out the search process to obtain results for sports foods supplements containing this substance.

Once the sample had been obtained, the investigation was redirected towards each of the web portals of the selected supplement brands (company’s website) in order to assemble the health claims used by advertisers, manufacturers or distributors. The process of obtaining each component of the sample was different depending on the web portal visited.

### Inclusion criteria

Supplements defined as ‘Creatine Monohydrate’ and for sale in Europe were part of the selected sample. In addition, supplements found on the selected web portals that declared some health claims, and provided information on the dosage of the product, were included in the sample.

### Exclusion criteria

Supplements that were not defined as ‘Creatine Monohydrate’ or that appeared multiple times within the search were excluded from the sample. Furthermore, supplements that did not declare any health claims, or that did not provide information on the dosage of the product, were also not part of the selected sample.

### Data extraction

Following the guidelines of content analysis, a questionnaire or analysis protocol was applied to the sample, allowing its quantitative and qualitative study. The variables included in said protocol were the following:

- Product name: the name of each of the sports foods supplements belonging to the sample was specified.
- Sports company: the brand of each of the CM supplements belonging to the sample was defined (Appendix 1).
- Dosage: the consumption (amount) recommended by the manufacturer, for each of the supplements.
- Health claims regarding CM: the health claims regarding CM present on the label of each of the sports foods supplements in the sample, as well as the information present on the website where the product was marketed.
- Degree of compliance: this refers to the extent to which the health claims regarding the CM present in the selected sample of sports foods supplements conformed to the health claims regarding this substance approved by European current legislation and current scientific evidence.
- Health claims modification: removal/modification of health claims made on the labelling of CM supplements so that they conformed more closely to the approved claims in Europe.

### Compliance with the legislation and scientific evidence

The commercial communications and the labelling of the selected sample were evaluated using the European regulation Commission Regulation (EU) No 432/2012^(20)^ and Commission Implementing Regulation (EU) 2017/672^(21)^ regarding the authorised health claims for CM described through the effect and cause-effect relationship by EFSA^(7,8)^ and EC^(20,21)^. In addition, the results were also compared with the documents from the IOC^(3)^, the International Society of Sports Nutrition (ISSN)^(22)^ and the AND^(16)^, together with the systematic review ‘Effects of Creatine Supplementation on Athletic Performance in Soccer Players: A Systematic Review and Meta-Analysis’^(23)^ to include the current scientific evidence.

## Results

**Fig. 1:**
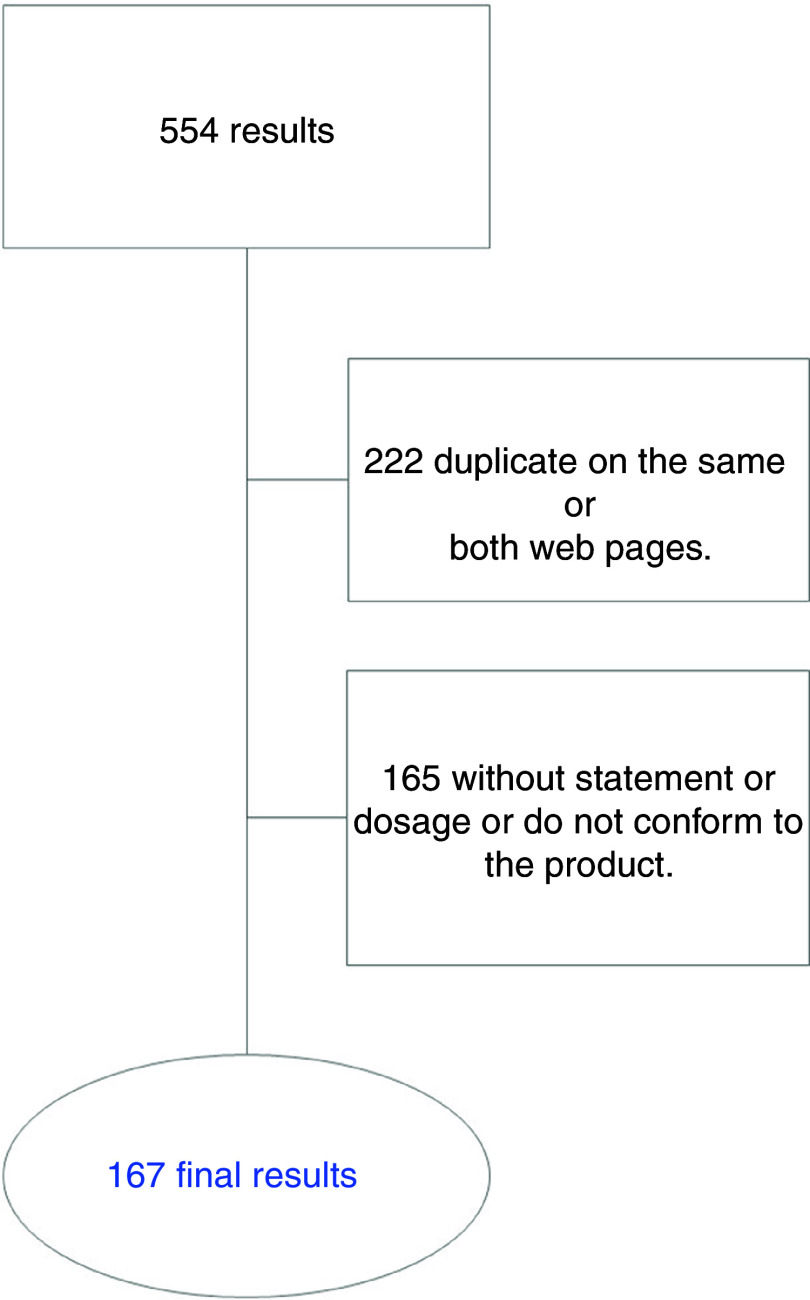
Flow diagram of the selection process for creatine monohydrate supplement

Of the 554 products identified in the initial search, 167 CM supplements, different from each other and belonging to different commercial brands, met the inclusion criteria and constituted the sample to be analysed; 222 were rejected for duplicity and another 165 for not including health claims or not conforming to the rest of the established criteria (Fig. 1).

For the sports foods supplements included in the sample, both the name of the product and the commercial brand, as well as the health claims presented and the dosage of each supplement, were specified. The most frequent dosage indicated in the sample of CM supplements studied was ‘5–5·9 g/d’, corresponding to 30·5 % of the total, while in 29·9 % of the cases the recommended dose was ‘3–3·9 g/d’.

### Current European Regulation and scientific evidence

Table 1 shows the information on effects, doses and applications of CM according to European regulation (EFSA and EC) and current scientific evidence.

**Table 1:** Effect and applications of the CM according to the European regulation and scientific evidence established by the reference institutions

	Sports involved	Dose	Ergogenic effects
European regulation			
EFSA and European Commission^(7)^	Improves physical performance in sport events with explosive activities especially of a repeated nature.	Daily intake of 3 g of creatine.	Improvement in exercise performance in sport events that require explosive, high-energy output activities especially of a repeated nature.
	Improvement in muscle strength and muscle function in adults over the age of 55 years who are engaged in regular resistance training. Beneficial effects are related with resistance exercise. Resistance training which allows an increase in the workload overtime should be performed at least three times per week for several weeks, at an intensity of at least 65–75 % of one repetition maximum.	In order to obtain the claimed effect, 3 g of creatine should be consumed daily.	Creatine intake improves muscle training capacity in higher intensities, which allows a higher muscle strength development.
Scientific evidence			
AND^(16)^	Improves performance of repeated bouts of high-intensity exercise with short recovery periods. - Direct effect on competition performance. - Enhanced capacity for training.	No data.	Increases creatine and phosphocreatine concentrations. May also have other effects such as enhancement of glycogen storage and direct effect on muscle protein synthesis.
IOC^(3)^	Can acutely enhance the performance of sports involving repeated high-intensity exercise (e.g. team sports), as well as the chronic outcomes of training programmes based on these characteristics (e.g. resistance or interval training), leading to greater gains in lean mass and muscular strength and power.	Loading phase: ~20 g/d (divided into four equal daily doses), for 5–7 d. Maintenance phase: 3–5 g/d (single dose) for the duration of the supplementation.	Supplementation increases muscle creatine stores, augmenting the rate of phosphocreatine re-synthesis, thereby enhancing short-term, high-intensity exercise capacity and the ability to perform repeated bouts of high-intensity effort.
ISSN^(9)^	Ergogenic aid for power/strength athletes to help them optimise training adaptations or athletes who need to sprint intermittently and recover during competition (e.g. American football, soccer, basketball, tennis, etc.).	Loading phase: ~0·3 g/kg/d of creatine monohydrate for 5–7 d. Maintenance phase: 3–5 g/d to maintain elevated stores. Cyclic dose: 3 g/d for 28 d to increase muscle creatine stores. 6 g/d for 12 weeks to enhance muscle volume and strength.	It increases the amount of muscle phosphocreatine, lean muscle mass, muscle strength, performance in sprints (aerobic capacity), power, training capacity and muscle diameter.
Effects of Creatine Supplementation on Athletic Performance in Soccer Players: A Systematic Review and Meta-Analysis^(20)^	Beneficial in anaerobic sports such as football.	Loading phase: 20–30 g/d, divided into 3–4 shots a day, for 6–7 d. Maintenance phase: 5 g/d for 9 weeks. Continued reduced dose: 3 mg/kg/d for 14 d.	It produces positive effects in physical performance tests related to anaerobic metabolism, especially with anaerobic power.

EFSA: European Food Safety Authority; AND: Academy of Nutrition and Dietetics; IOC: International Olympic Committee; ISSN: International Society of Sports Nutrition.

### Health claims

Table 2 establishes a percentage distribution of each health property declaration found in the sample of supplements and, in addition, compares the proposed dosages and the type of health property declaration indicated by the manufacturer for each CM sports supplement.

**Table 2:** Distribution of product doses according to the health claims and their reasons of adequacy

	Total supplements in which this health claim is given		Doses	Supplements in which this dosage is given for this health claim		Reason of adequacy*
Health claim	*n*	%		*n*	%	
Improves strength	70	41·9 %	Less than 3 g/d	5	7·1 %	2
			3–3·9 g/d	10	14·3 %	3
			4–4·9 g/d	3	4·3 %	3
			5–5·9 g/d	28	40 %	3
			6–6·9 g/d	6	8·6 %	3
			More than or equal to 7 g/d	1	1·4 %	3
			Dose by ranges	6	8·6 %	3
			Doses per loading and maintenance phases	11	15·7 %	3
Increases muscle mass	78	46·7 %	Less than 3 g/d	6	7·7 %	3
			3–3·9 g/d	10	12·8 %	3
			4–4·9 g/d	3	3·8 %	3
			5–5·9 g/d	29	37·2 %	3
			6–6·9 g/d	5	6·4 %	3
			More than or equal to 7 g/d	2	2·6 %	3
			Dose by ranges	7	9 %	3
			Doses per loading and maintenance phases	16	20·5 %	3
Promotes cellular hydration	13	7·8 %	3–3·9 g/d	3	23·1 %	1
			5–5·9 g/d	5	38·5 %	1
			Dose by ranges	3	23·1 %	1
			Doses per loading and maintenance phases	2	15·4 %	1
Enhances physical performance	122	73·1 %	Less than 3 g/d	9	7·4 %	2
			3–3·9 g/d	42	34·4 %	3 and 4
			4–4·9 g/d	2	1·6 %	2
			5–5·9 g/d	36	29·5 %	2
			6–6·9 g/d	7	5·7 %	2
			More than or equal to 7 g/d	2	1·6 %	2
			Dose by ranges	11	9 %	2
			Doses per loading and maintenance phases	13	10·7 %	2
Improves recovery, prevents muscle fatigue or promotes muscle regeneration	52	31·1 %	Less than 3 g/d	7	13·5 %	1
			3–3·9 g/d	7	13·5 %	1
			4–4·9 g/d	2	3·8 %	1
			5–5·9 g/d	17	32·7 %	1
			6–6·9 g/d	2	3·8 %	1
			More than or equal to 7 g/d	1	1·9 %	1
			Dose by ranges	4	7·7 %	1
			Doses per loading and maintenance phases	12	23·1 %	1
Supports weight loss	2	1·2 %	Less than 3 g/d	1	50 %	1
			3–3·9 g/d	1	50 %	1
Increases physical resistance	20	12 %	Less than 3 g/d	3	15 %	1
			3–3·9 g/d	4	20 %	1
			5–5·9 g/d	6	30 %	1
			6–6·9 g/d	2	10 %	1
			Dose by ranges	1	5 %	1
			Doses per loading and maintenance phases	4	20 %	1
Increases power	38	22·8 %	Less than 3 g/d	1	2·6 %	2
			3–3·9 g/d	5	13·2 %	3
			4–4·9 g/d	1	2·6 %	3
			5–5·9 g/d	16	42·1 %	3
			6–6·9 g/d	3	7·9 %	3
			More than or equal to 7 g/d	2	5·3 %	3
			Dose by ranges	3	7·9 %	3
			Doses per loading and maintenance phases	7	18·4 %	3
Enhances physical performance in adults over the age of 55	2	1·2 %	3–3·9 g/d	1	50 %	3
			5–5·9 g/d	1	50 %	2
Improves energy availability	23	13·8 %	Less than 3 g/d	1	4·3 %	1
			3–3·9 g/d	4	17·4 %	1
			5–5·9 g/d	9	39·1 %	1
			6–6·9 g/d	2	8·7	1
			Dose by ranges	1	4·3 %	1
			Doses per loading and maintenance phases	6	26·1 %	1
Promotes the replacement of creatine stores	3	1·8 %	Less than 3 g/d	2	66·7 %	3
Replenishes muscle glycogen stores	2	1·2 %	Less than 3 g/d	1	50 %	3
			Dose by ranges	1	50 %	3
Improves defenses or promotes greater protection	2	1·2 %	3–3·9 g/d	1	50 %	1
			Doses per loading and maintenance phases	1	50 %	1
Promotes gastric emptying	1	0·6 %	Doses per loading and maintenance phases	1	100 %	1
Increases speed	1	0·6 %	5–5·9 g/d	1	100 %	1
Improves brain capacities	2	1·2 %	3–3·9 g/d	1	50 %	1
			5–5·9 g/d	1	50 %	1
Promotes cardiovascular health	2	1·2 %	Dose by ranges	1	50 %	1
			Doses per loading and maintenance phases	1	50 %	1
Improves blood glucose	1	0·6 %	Dose by ranges	1	100 %	1
Helps the growth of bones and cartilage	1	0·6	3–3·9 g/d	1	100 %	1

* Reason: Number 1: Cause: it does not conform to the approved health claims about the CM; Proposal to modify: delete health claim. Number 2: Cause: it conforms to approved health claims but does not meet the recommended dosage of the product; Proposal to modify: modify product dosage protocol to 3 grams. Number 3: Cause: it conforms to the approved health claims and the correct dose established but does not specify the type of exercise performed; Proposal to modify: the claim effects must be specified for ‘sport events that require explosive, high-energy output activities especially of a repeated nature’^(7)^. Number 4: Cause: it conforms to the approved health claim, specifying the appropriate recommended dose of the product and the effects of the supplement; Proposal to modify: Not modify or remove the health claim as it fits properly.

The health claim most often described for the CM supplements was ‘Enhances physical performance’, appearing in 73·1 % of the supplements in the sample. At a distance, this was followed by ‘Increases muscle mass’ (46·7 %), ‘Improves strength’ (41·9 %), ‘Improves recovery, prevents muscle fatigue or promotes muscle regeneration’ (31·1 %), ‘Increases power’ (22·8 %), ‘Improves energy availability’ (13·8 %), ‘Increases physical resistance’ (12·0 %) and ‘Promotes cellular hydration’ (7·8 %). Less frequent health claims were ‘Supports weight loss’, ‘Enhances physical performance in adults over the age of 55’, ‘Promotes the replacement of creatine stores’, ‘Replenishes muscle glycogen stores’, ‘Improves defenses or promotes greater protection’, ‘Improves brain capacities’ and ‘Promotes cardiovascular health’. In addition, there were health claims that only appeared in a single product among the sample of sports foods supplements, such as ‘Promotes gastric emptying’, ‘Increases speed’, ‘Improves blood glucose’ and ‘Helps the growth of bones and cartilage’.

### Dosage and health claims

For the statement ‘Improves strength’, a dosing protocol for loading and maintenance was indicated in most CM supplements; such a protocol was also specified for the only product that declared ‘Promotes gastric emptying’ as a CM health effect.

In the case of the health claims ‘Increases muscle mass’, ‘Promotes cellular hydration’, ‘Improves recovery, prevents muscle fatigue or promotes muscle regeneration’, ‘Increases physical resistance’, ‘Increases power’ and ‘Improves energy availability’, a dosage of 5–5·9 g/d of the CM supplement was recommended for most supplements. A similar dose was also indicated for the only supplement in the sample with the statement ‘Increases speed’.

For the products with the health claim ‘Enhances physical performance’, a dose of 3–3·9 g/d was proposed; likewise for the only supplement for which ‘Helps the growth of bones and cartilage’ was affirmed.

For the health claim ‘Promotes the replacement of creatine stores’, the manufacturers of said supplements indicated a dose below 3 g/d in two of the three CM supplements. For the only supplement for which ‘Improves blood glucose’ was declared, the indicated dosage was 5 to 10 g of product per day.

The rest of the health claims ‘Supports weight loss’, ‘Enhances physical performance in adults over the age of 55’, ‘Replenishes muscle glycogen stores’, ‘Improves defenses or promotes greater protection’, ‘Improves brain capacities’ and ‘Promotes cardiovascular health’ appeared for only two supplements in the sample, with the two dosages being different in each case.

Table 2 also indicates the adequacy of the declarations for the CM products in the sample. Only the affirmation ‘Enhances physical performance’, for some of the supplements with a recommended dose of 3–3·9 g/d, conformed to the approved guidelines which specified the appropriate recommended dose of the product and the effects produced by it; hence, a recommendation is proposed not to modify or eliminate the statement. The reasons for considering the declarations of the CM products to be adequate or non-adequate, as well as the modifications that should be made, were made in agreement with European regulation documents (Table 1).

For the health claims ‘Improves strength’, ‘Increases muscle mass’, ‘Enhances physical performance’, ‘Increases power’, ‘Enhances physical performance in adults over the age of 55’, ‘Promotes the replacement of creatine stores’ and ‘Replenishes muscle glycogen stores’, it can be verified that they conformed to the health claims of the institutions involved. In most CM supplements, the appropriate dosages were shown; however, all of them should be modified because they did not specify the type of exercise to which the claimed effects relate.

Also, the affirmations ‘Promotes cellular hydration’, ‘Improves recovery, prevents muscle fatigue or promotes muscle regeneration’, ‘Supports weight loss’, ‘Increases physical resistance’, ‘Improves energy availability’, ‘Improves defenses or promotes greater protection’, ‘Promotes gastric emptying’, ‘Increases speed’, ‘Improves brain capacities’, ‘Promotes cardiovascular health’, ‘Improves blood glucose’ and ‘Helps the growth of bones or cartilage’ did not adequately conform to the standards established by the relevant institutions.

## Discussion

Only 42 of 167 supplements from the total study sample fully or partially complied with what was established by the EFSA and EC; thus, the health claim ‘Enhances physical performance’ with a dose of 3–3·9 g/d corresponded to 25 % of the study sample. The most common dosage recommended among the products in the sample (for 30·5 % of the total number) was 5–5·9 g/d, followed by the minimum dose recommended by the institutions of 3–3·9 g/d (for 29·9 % of the supplements). The health effect that was most frequently claimed – for 73·1 % of the products – was ‘Enhances physical performance’, whereas ‘Enhances physical performance in adults over the age of 55’ was only stated in two supplements from the sample. The use of CM supplements in sports is part of an attempt to improve physical performance in certain sports modalities. These supplements are marketed with distinct brand names by many companies, highlighting the effects they produce and the dosage recommended by the manufacturer. In some cases, these data do not conform to what has been established by various institutions – such as the EFSA, AND, IOC and ISSN – or by the latest reviews based on scientific evidence^(3,7,8,16,22,23)^.

### Health claims and proposed dosages

Currently, athletes are exposed to a significant volume of commercial communications including claims about improvements in performance and recovery produced by a wide range of products, including sports foods supplements. Legislation on nutrition and health claims, as well as claims of ergogenic effects, requires that they be supported by scientific evidence and cannot mislead consumers by creating false expectations by exaggerating the performance-enhancing ability of a certain product^(24,25)^.

Athletes sometimes obtain scarce and/or confusing information on the use of these products, to which we can add the lack of knowledge of the athletes on both aspects related to general nutrition and the specific needs of the practice of sports, which some studies highlight^(26)^. This lack of knowledge can be exacerbated if there are erroneous beliefs about eating habits confounded by friends, family, coaches, advertising, etc.^(27)^.

Within the sample of supplements examined in this study, 19 different health effects were claimed by the manufacturers. Of these, only seven declarations fully or partially complied with that established by the relevant institutions, consensus documents or scientific evidence. These declarations are approved at a European level by the EFSA and the EC, the latter having specific legislation to regulate these products^(9,15,28)^. As indicated, only in the case of the claims ‘Improves strength’, ‘Increases muscle mass’, ‘Enhances physical performance’, ‘Increases power’, ‘Enhances physical performance in adults over the age of 55’, ‘Promotes the replacement of creatine stores’ and ‘Replenishes muscle glycogen stores’, their adequacy was confirmed. It should be noted that the effects declared for CM appear with minimum doses of 3 g/d of the product.

### Action and proposals to deal with incomplete, inaccurate or confusing health claims

From the point of view of the advertising and marketing of food products, the EFSA is involved in food safety in the context of public health at the European level^(9)^. The administrative and judicial actions that derive from the aforementioned regulatory framework are completed with the activity of organisations such as the Association of Communication Users (AUC), to which any user can report inappropriate advertising content that they consider not suitable for the public or the advertising object, and Autocontrol, an independent self-regulatory body for commercial communications. Some studies show that some sports foods supplements are not based on correct scientific references^(26)^. The study by Molinero *et al.* points out that 52·8 % of the sports foods supplements analysed did not provide scientific references in accordance with the statements attributed to them^(29)^. In this study, some of the health claims were not in agreement with the scientific evidence^(3,10,16,22,23)^, such as ‘Helps the growth of bones and cartilage’, ‘Supports weight loss’ or ‘Promotes cardiovascular health’.

It is therefore recommended that the European institutions and policy decision-makers work together to achieve their objectives by focusing on advertising that is suitable for all audiences and appropriate to the product or service offered^(27)^. To establish a policy recommendation and to move this process forward, an appropriate institutional setting is needed. Consumer protection provisions should promote greater levels of policy development, regulatory enforcement and consumer education^(9)^.

### Advertising fraud cases

Fraudulent sports foods supplements-related situations often occur. The aforementioned organisations have had to act on multiple occasions, serving as a tool for citizens to complain to the advertiser. Two cases about incorrect health claims (advertising fraud) in sports foods are Amino X and Vitargo. In the Amino X case, effects of the product that were not real were cited, leading to consumer fraud. For Vitargo, the existence of nutrients in the product was declared, but certain criteria for their concentrations were not met, and it was implied that the amounts present were higher than those actually contained within the product^(30)^.

This type of fraud has also been observed in food. The Foodwatch study, conducted in the Netherlands and Germany, highlighted the use of claims of health-enhancing properties or the presence of high concentrations of certain nutrients as a marketing tool for unhealthy foods^(31)^. In addition to incorrect health claims, differences between the labelling and composition of CM can also be found^(32)^, and prohibited or undeclared substances may even be present^(3)^.

### Study limitations

The search for the sample of products was carried out in April 2019. Thus, newer supplements may be available in the search portals used that are not included in the present results.

Another of the difficulties of the study was the variability of the results from the search portals, as well as the existence of products that did not offer the information required for the study.

This work also highlights the multitude of health claims made by manufacturers or advertisers, in some cases presenting very confusing information.

## Conclusions

The health claims made for sports foods supplements must completely conform to the criteria established by European legislation and must be in agreement with scientific evidence. Sports foods supplements fraud is found in various forms within the advertising and marketing of food, strongly affecting consumers. Lastly, ensuring the quality of food advertising must be the job of advertisers, consumers and public health or sports policies and must reflect the rights of consumers defined by current regulations.

## Supplementary material

For supplementary material accompanying this paper visit https://doi.org/10.1017/S1368980020005121. click here to view supplementary material
