# Supplementary material for: Analysis of health claims regarding creatine monohydrate present in commercial communications for a sample of European sports foods supplements
Source: Public Health Nutr. 2021 Jan 20;24(4):632–40. doi: 10.1017/S1368980020005121 (PMC11574825; doi:10.1017/S1368980020005121)
Supplement: Supplementary file 1 [file S1368980020005121sup001.pdf]

Appendix 1. Supplementation company and health claims on their products

HEALTH CLAIM	SUPPLEMENTATION COMPANY
**Improves strength**	▪ HSN Raw<br>▪ Natural Health UP<br>▪ ON Optimum Nutrition (3 products)<br>▪ Crown Sport Nutrition<br>▪ Amix (2 products)<br>▪ Quamtrax<br>▪ MTX Active Nutrition<br>▪ Probulus<br>▪ Xcore Nutrition<br>▪ Joe Weider Victory<br>▪ Master ZX<br>▪ Evlution Nutrition<br>▪ Muscle Force<br>▪ Tri-Nine<br>▪ Big Protein<br>▪ Creatine-Labs<br>▪ Best Protein<br>▪ Aminostar<br>▪ 4+Nutrition<br>▪ American Supplement<br>▪ MP Muscle Pharm<br>▪ Heal Secrets<br>▪ ASN<br>▪ Hypertrophy<br>▪ Record Nutrition<br>▪ Scitec Nutrition (2 products)<br>▪ Firm Foods<br>▪ Olimp Sport Nutrition<br>▪ Okygen<br>▪ Universal Nutrition<br>▪ MuscleTech<br>▪ Big Man<br>▪ Gold Nutrition<br>▪ Trec Nutrition (2 products)<br>▪ QXN<br>▪ Black Line<br>▪ EU Nutrition<br>▪ MET-Rx (2 products)<br>▪ QNT<br>▪ Applied Nutrition<br>▪ Nutrytec (2 products)<br>▪ MP Essentials<br>▪ Natural Diet<br>▪ OstroVit<br>▪ MHP<br>▪ Nutrex<br>▪ Devotika<br>▪ Scientiffic Nutrition<br>▪ ActivLab (2 products)<br>▪ MAN Sports<br>▪ Vitobest NEO<br>▪ GAT Sport

	▪ AllMax Nutrition<br>▪ Haya Labs<br>▪ Life PRO<br>▪ Genetix Labs<br>▪ OWN PWR<br>▪ EAFit<br>▪ Vitobest<br>▪ FitWhey<br>▪ Elite PRO<br>▪ Onnit<br>▪ BPI Sports<br>▪ Evogen<br>▪ Sheer Strength Labs<br>▪ BioChem<br>▪ Named Sport
**Increases muscle mass**	▪ HSN Raw<br>▪ Natural Health UP<br>▪ ON Optimum Nutrition (3 produts)<br>▪ Qualnat<br>▪ Crown Sport Nutrition<br>▪ Amix (2 produts)<br>▪ Starlabs Nutrition<br>▪ MTX Active Nutrition<br>▪ Xcore Nutrition<br>▪ Abs<br>▪ Evlution Nutrition<br>▪ JeaKen<br>▪ Muscle Force<br>▪ Tri-Nine<br>▪ FSA Sports Nutrition<br>▪ Creatine-Labs<br>▪ Best Protein<br>▪ Aminostar<br>▪ B4Fit<br>▪ Vitobest (2 produts)<br>▪ Just Aid<br>▪ MRM<br>▪ Multipower<br>▪ MP Muscle Pharm<br>▪ Heal Secrets<br>▪ Beverly Nutrition<br>▪ Lamberts<br>▪ ASN<br>▪ Hypertropy<br>▪ Record Nutrition<br>▪ Scitec Nutrition<br>▪ Firm Foods<br>▪ Okygen<br>▪ Nutrend<br>▪ Now Sports<br>▪ Muscle Tech<br>▪ Pure Nutrition<br>▪ Big Man<br>▪ Gold Nutrition<br>▪ Black Line<br>▪ GoNutrition

	▪ Cellucor<br>▪ EU Nutrition<br>▪ MET-Rx (2 produts)<br>▪ QNT<br>▪ Applied Nutrition<br>▪ Nutrytec (3 produts)<br>▪ MP Essentials<br>▪ Natural Diet<br>▪ OstroVit<br>▪ Nutrex<br>▪ Devotika<br>▪ Scientiffic Nutrition<br>▪ Iron Supplement<br>▪ ActivLab (2 produts)<br>▪ Vitobest NEO<br>▪ AllMax Nutrition (2 produts)<br>▪ EAFit<br>▪ Trec Nutrition<br>▪ FitWhey<br>▪ Onnit<br>▪ SCI-MX<br>▪ BPI Sports<br>▪ Evogen<br>▪ Sheer Strength Labs<br>▪ Named Sport
**Promotes cellular hydration**	▪ HSN Raw<br>▪ Xcore Nutrition<br>▪ Scitec Nutrition (2 produts)<br>▪ Universal Nutrition<br>▪ Muscle Tech<br>▪ Cellucor<br>▪ Natural Diet<br>▪ Amix<br>▪ Vitobest NEO<br>▪ 3XL Nutrition<br>▪ Haya Labs<br>▪ Trec Nutrition
**Enhances physical performance**	▪ HSN Raw<br>▪ Vegavero Sport<br>▪ SanaExpert<br>▪ Natural Health UP<br>▪ MyProtein (6 produts)<br>▪ Scitec Nutrition (3 produts)<br>▪ Qualnat<br>▪ Amix<br>▪ BioTech USA<br>▪ Prozis (5 produts)<br>▪ Xtraze<br>▪ Starlabs Nutrition<br>▪ The Protein Works<br>▪ Universal Nutrition (2 produts)<br>▪ Xcore Nutrition<br>▪ Joe Weider Victory<br>▪ Olimp Sport Nutrition (6 produts)<br>▪ Quamtrax<br>▪ Abs

	▪ Evlution Nutrition<br>▪ Trec Nutrition (3 produts)<br>▪ SimplyGo<br>▪ Yamamoto Nutrition (2 produts)<br>▪ HSN Store<br>▪ LEAN Active<br>▪ JeaKen<br>▪ MyVitamins<br>▪ Infisport<br>▪ Amando Pérez<br>▪ Muscle Force<br>▪ Nu3<br>▪ Tri-Nine<br>▪ FSA Sports Nutrition<br>▪ Protein Buzz<br>▪ Big Protein<br>▪ Jarrow Formulas<br>▪ Aminostar<br>▪ Bodybuilding Warehouse<br>▪ 4+Nutrition<br>▪ Just Aid<br>▪ Multipower<br>▪ Nutrend (3 produts)<br>▪ MP Muscle Pharm<br>▪ Dymatize<br>▪ Heal Secrets<br>▪ Beverly Nutrition<br>▪ NutriSport<br>▪ ESN<br>▪ Hypertrophy<br>▪ Reflex Nutrition<br>▪ Tropicana<br>▪ Hero Tech Nutrition<br>▪ Ares Nutrition<br>▪ Best Body Nutrition<br>▪ Raw Physique<br>▪ Grenade<br>▪ Now Sports<br>▪ Pure Nutrition<br>▪ Isopure<br>▪ Black Line<br>▪ Aptonia (2 produts)<br>▪ MET-Rx (2 produts)<br>▪ Applied Nutrition<br>▪ Etixx<br>▪ Nutrytec (3 produts)<br>▪ Efectiv Nutrition<br>▪ MP Essentials<br>▪ Natural Diet<br>▪ OstroVit<br>▪ MHP<br>▪ Nutrex<br>▪ Scientiffic Nutrition<br>▪ Iron Supplement<br>▪ ActivLab<br>▪ MAN Sports

	▪ Muscle Tech<br>▪ GAT Sport<br>▪ Bulk Powders<br>▪ Galvanize Nutrition<br>▪ Everlast (2 produts)<br>▪ QNT<br>▪ 3XL Nutrition<br>▪ AllMax Nutrition<br>▪ Haya Labs<br>▪ Genetix Labs<br>▪ OWN PWR (2 produts)<br>▪ Vitobest<br>▪ Elite PRO<br>▪ Motion Nutrition<br>▪ Reflex XFT<br>▪ Onnit<br>▪ Named Sport
**Helps the growth of bones and cartilage**	▪ Fairvital
**Supports weight loss**	▪ Natural Health UP<br>▪ Record Nutrition
**Increases physical resistance**	▪ Qualnat<br>▪ Probulus<br>▪ Best Protein<br>▪ American Supplement<br>▪ Multipower<br>▪ Record Nutrition<br>▪ Hero Tech Nutrition<br>▪ Okygen<br>▪ Universal Nutrition<br>▪ Muscle Tech<br>▪ Black Line<br>▪ GoNutrition<br>▪ Cellucor<br>▪ EU Nutrition<br>▪ Applied Nutrition<br>▪ OstroVit<br>▪ ActivLab<br>▪ Haya Labs<br>▪ Vitobest<br>▪ Named Sport
**Increases power**	▪ ON Optimum Nutrition (2 produts)<br>▪ Crown Sport Nutrition<br>▪ Amix<br>▪ Starlabs Nutrition<br>▪ Probulus<br>▪ Master ZX<br>▪ LEAN Active<br>▪ Tri-Nine<br>▪ Big Protein<br>▪ 4+Nutrition<br>▪ B4Fit<br>▪ Vitobest (2 produts)<br>▪ American Supplement<br>▪ MRM<br>▪ MP Muscle Pharm

	▪ Scitec Nutrition (2 produts)<br>▪ Firm Foods<br>▪ Big Man<br>▪ QXN<br>▪ Cellucor<br>▪ EU Nutrition<br>▪ MET-Rx (2 produts)<br>▪ Applied Nutrition<br>▪ MP Essentials<br>▪ Nutrex<br>▪ Devotika<br>▪ Nutrytec<br>▪ MAN Sports<br>▪ Vitobest NEO<br>▪ OWN PWR<br>▪ EAFit<br>▪ Trec Nutrition<br>▪ Elite PRO<br>▪ BPI Sports<br>▪ Evogen<br>▪ Sheer Strength Labs
**Enhances physical performance in adults over the age of 55**	▪ SimplyGo<br>▪ HSN Store
**Improves energy availability**	▪ Vegavero Sport<br>▪ Qualnat<br>▪ BSN<br>▪ Universal Nutrition (2 produts)<br>▪ Xcore Nutrition<br>▪ JeaKen<br>▪ Nu3<br>▪ Too Shaped<br>▪ Best Protein<br>▪ Vitobest (2 produts)<br>▪ American Supplement<br>▪ Just Aid<br>▪ Olimp Sport Nutrition<br>▪ Record Nutrition<br>▪ Scitec Nutrition (2 produts)<br>▪ Firm Foods<br>▪ Okygen<br>▪ QXN<br>▪ Cellucor<br>▪ Nutrytec<br>▪ Scientiffic Nutrition<br>▪ MAN Sports<br>▪ 3XL Nutrition<br>▪ Named Sport
**Promotes the replacement of creatine stores**	▪ BSN<br>▪ Applied Nutrition<br>▪ Vitobest
**Replenishes muscle glycogen stores**	▪ MTX Active Nutrition<br>▪ Scitec Nutrition
**Improves defenses or promotes greater protection**	▪ Fairvital<br>▪ Muscle Tech
**Promotes gastric emptying**	▪ Scientiffic Nutrition

**Increases speed**	▪ MTX Active Nutrition<br>▪ Big Protein<br>▪ Hero Tech Nutrition<br>▪ Scientiffic Nutrition<br>▪ Vitobest NEO
**Improves brain capacities**	▪ Fairvital<br>▪ Tri-Nine
**Promotes cardiovascular health**	▪ Scitec Nutrition<br>▪ Scientiffic Nutrition
**Improves blood glucose**	▪ Scitec Nutrition
**Improves recovery, prevents muscle fatigue or promotes muscle regeneration**	▪ HSN Raw<br>▪ Natural Health UP<br>▪ Qualnat<br>▪ Crown Sport Nutrition<br>▪ Amix<br>▪ Quamtrax<br>▪ MTX Active Nutrition<br>▪ Probulus<br>▪ Universal Nutrition (2 produts)<br>▪ Joe Weider Victory<br>▪ Big Protein<br>▪ Creatine-Labs<br>▪ Aminostar<br>▪ B4Fit<br>▪ Vitobest (2 produts)<br>▪ American Supplement<br>▪ Just Aid<br>▪ MRM<br>▪ Olimp Sport Nutrition<br>▪ NutriSport<br>▪ Lamberts<br>▪ Weider (2 produts)<br>▪ Scitec Nutrition<br>▪ Firm Foods<br>▪ Okygen<br>▪ Muscle Tech<br>▪ QXN<br>▪ Black Line<br>▪ GoNutrition<br>▪ EU Nutrition<br>▪ MET-Rx (2 produts)<br>▪ Applied Nutrition<br>▪ Nutrytec (2 produts)<br>▪ Scientiffic Nutrition<br>▪ Vitobest NEO<br>▪ ActivLab<br>▪ GAT Sport<br>▪ AllMax Nutrition<br>▪ Haya Labs<br>▪ Life PRO<br>▪ EAFit<br>▪ Trec Nutrition<br>▪ BPI Sports<br>▪ Sheer Strength Labs<br>▪ Named Sport
